# Genome-Wide Association Study in Pseudo-F_2_ Populations of Switchgrass Identifies Genetic Loci Affecting Heading and Anthesis Dates

**DOI:** 10.3389/fpls.2018.01250

**Published:** 2018-09-13

**Authors:** Megan Taylor, Carl-Erik Tornqvist, Xiongwei Zhao, Paul Grabowski, Rebecca Doerge, Jianxin Ma, Jeffrey Volenec, Joseph Evans, Guillaume P. Ramstein, Millicent D. Sanciangco, C. Robin Buell, Michael D. Casler, Yiwei Jiang

**Affiliations:** ^1^Department of Agronomy, Purdue University, West Lafayette, IN, United States; ^2^U.S. Department of Energy, Great Lakes Bioenergy Research Center and Department of Agronomy, University of Wisconsin-Madison, Madison, WI, United States; ^3^Maize Research Institute, Sichuan Agricultural University, Chengdu, China; ^4^U.S. Dairy Forage Research Center, United States Department of Agriculture-Agricultural Research Service, Madison, WI, United States; ^5^Department of Biology and Department of Statistics, Mellon College of Science, Carnegie Mellon University, Pittsburgh, PA, United States; ^6^U.S. Department of Energy, Great Lakes Bioenergy Research Center and Department of Plant Biology, Michigan State University, East Lansing, MI, United States

**Keywords:** GWAS, candidate gene, heading, flowering time, *Panicum virgatum*

## Abstract

Switchgrass (*Panicum virgatum*) is a native prairie grass and valuable bio-energy crop. The physiological change from juvenile to reproductive adult can draw important resources away from growth into producing reproductive structures, thereby limiting the growth potential of early flowering plants. Delaying the flowering of switchgrass is one approach by which to increase total biomass. The objective of this research was to identify genetic variants and candidate genes for controlling heading and anthesis in segregating switchgrass populations. Four pseudo-F_2_ populations (two pairs of reciprocal crosses) were developed from lowland (late flowering) and upland (early flowering) ecotypes, and heading and anthesis dates of these populations were collected in Lafayette, IN and DeKalb, IL in 2015 and 2016. Across 2 years, there was a 34- and 73-day difference in heading and a 52- and 75-day difference in anthesis at the Lafayette and DeKalb locations, respectively. A total of 37,901 single nucleotide polymorphisms obtained by exome capture sequencing of the populations were used in a genome-wide association study (GWAS) that identified five significant signals at three loci for heading and two loci for anthesis. Among them, a homolog of FLOWERING LOCUS T on chromosome 5b associated with heading date was identified at the Lafayette location across 2 years. A homolog of ARABIDOPSIS PSEUDO-RESPONSE REGULATOR 5, a light modulator in the circadian clock associated with heading date was detected on chromosome 8a across locations and years. These results demonstrate that genetic variants related to floral development could lend themselves to a long-term goal of developing late flowering varieties of switchgrass with high biomass yield.

## Introduction

Switchgrass is a C4 perennial bioenergy and forage grass. Switchgrass has been chosen as a herbaceous species for biofuel feedstock development due to its adaptation across climates, high biomass yields, tolerance to marginal conditions, and low input requirements. Switchgrass consists of upland and lowland ecotypes. Upland types are commonly tetraploid (2n = 4x = 36), but can be octoploid (2n = 8x = 72) or hexaploid (2n = 6x = 54 chromosomes), whereas lowland ecotypes are typically tetraploids (Narasimhamoorthy et al., 2008). However, switchgrass displays disomic inheritance at the tetraploid ploidy level (Casler, 2012). Upland ecotypes are adapted to northern climates with earlier flowering times and producing low biomass, while lowland ecotypes are adapted to southern climates with later flowering times and production of high biomass (Casler, 2012). Northern accessions of switchgrass reach peak biomass at flowering time, about 6–8 weeks before killing frost. Delaying flowering by 4–5 weeks can increase biomass yield by 30 to 50% (Casler, 2012). Theoretically, a delay in flowering time could be achieved by the use of either upland or lowland ecotypes. For upland ecotypes, this would involve intensive selection for late flowering within adapted germplasm. For lowland ecotypes, this would involve intensive selection for cold tolerance and adaptability within populations from southern latitudes that are already 4–6 weeks later in flowering compared to northern populations (Casler and Boe, 2003; Casler et al., 2004). Switchgrass requires short days to flower. When short-day grasses are grown in long day conditions, tillers remain vegetative for a longer period of time, resulting in more phytomers and delayed and flowering (Van Esbroeck et al., 2003).

Plants possess an internal biological clock, the circadian clock, which responds to day length and sends signals altering the plant for upcoming seasonal changes (Nuñez and Yamada, 2017). The transition from vegetative to reproductive phase causes a variety of signals and pathways to be activated in plant species. Several pathways regulate flowering time, including photoperiod, circadian clock, vernalization, autonomous, hormone and the aging (Khan et al., 2014; Blumel et al., 2015; Tornqvist et al., 2017). Photoperiod and circadian clock pathways are conserved in most plant species and work jointly using diurnal rhythms of the circadian clock gene expression to induce expression of downstream genes dependent on the light cycle (Song et al., 2010; Johansson and Staiger, 2015). Flowering in *Arabidopsis thaliana* relies on the day-length-dependent induction of FLOWERING LOCUS T (FT), encoding Florigen, a well-characterized protein which is synthesized in the leaf tissue and moves to the shoot apex to initiate floral development (Song et al., 2013). The expression of FT is primarily regulated by the transcriptional activator CONSTANS (CO), whose activity is tightly controlled by circadian clock and light (Song et al., 2013). Upon arriving in the meristem, FT binds to FLOWERING LOCUS D (FD), a bZIP transcription factor to form the FT-FD complex, regulating meristem identity genes (Wickland and Hanzawa, 2015). APETALA (AP1) and FRUITFULL (FUL) are meristem identity genes that are directly activated by the FT-FD complex and signal the primordia to begin making reproductive, non-vegetative, structures in *A. thaliana* (Blumel et al., 2015). In switchgrass, *PvFT1, PvAPL1-3*, and *PvSL1,2* have been identified as critical regulatory factors controlling floral initiation and development of floral organs (Niu et al., 2016). Overexpression of *PvFT1* was found to induce extremely early flowering in switchgrass (Niu et al., 2016).

Vernalization is required for flowering in some plant species (Kim et al., 2009), but the relevant mechanisms are not conserved. In *A. thaliana*, the vernalization pathway relies on the interaction of FLOWERING LOCUS C (FLC) with other regulators to control flowering development (Khan et al., 2014). FLC prevents flowering by preventing the transcriptional activation of SUPPRESSOR OF OVEREXPRESSION OF CO1 (SOC1) and FT by interfering with their chromatin (Helliwell et al., 2006). This affects the ability of the photoperiod and circadian clock pathway to activate floral integrators. VERNALIZATION1 (VRN1), VERNALIZATION2 (VRN2), and VERNALIZATION3 (VRN3) are three important genes that control the vernalization pathway in winter wheat (*Triticum aestivum*) and barley (*Hordeum vulgare*) varieties (Kim et al., 2009). VRN1 encodes a MADS-Box transcription factor that is similar to AP1 and FUL meristem identity genes (Trevaskis et al., 2003). VRN2 acts as a floral repressor by blocking VRN3, the cereal homolog to FT (Yan et al., 2006). VRN1 also has a dual role in the downstream promotion of flowering using VRN3 and in cold-induced upstream repression of VRN2 (Kim et al., 2009). The dual role of VRN1 in the cereal vernalization pathway creates a positive flowering feedback loop that is not found in *A. thaliana*. In addition, the repression of VRN1 in *Brachypodium distachyon* using REPRESSOR OF VERNALIZATION1 (RVR1) is required for vernalization (Woods et al., 2017), indicating that a variety of pathways control vernalization within grasses and cereals. Several grass species do not require vernalization, including switchgrass. This may be related to flowering time being in the summer and autumn, but not in spring (Michaels and Amasino, 2000).

Homologs of FT, FTLIKE9/10 (FTL9/10), and AGAMOUS-LIKE 16 (AGL16) associated with heading date have been identified through GWAS in natural populations of switchgrass originating primarily from northern latitudes of its range (Grabowski et al., 2017). In addition, quantitative trait loci (QTL) for heading and anthesis dates have been detected in a pseudo-F_2_ switchgrass population of 342 genotypes (Tornqvist et al., 2018). While phenotypic variation of flowering time in switchgrass is largely driven by the latitude of genotype origin (McMillan, 1965; Casler and Boe, 2003), genetic mechanisms underlying flowering time are not yet well understood in this species. We have developed four tetraploid switchgrass mapping populations by creating two reciprocal pseudo-F_2_ crosses derived from an upland, early flowering and a lowland late flowering ecotype. Using phenotypic data for heading and anthesis dates and genotypic data based on exome capture sequencing, we conducted GWAS to identify genetic loci and candidate genes affecting heading and anthesis dates across two geographical locations. The results will provide insights into genetic mechanisms of flowering time and could assist in developing late flowering varieties of switchgrass with high biomass yield.

## Materials and Methods

### Plant Materials and Planting Design

The four crosses represent second-generation crosses, originating from plant B901 of the ‘Ellsworth’ lowland population and plant S041 from the ‘Summer’ upland population. The initial cross was made in 2012 at the U.S. Dairy Forage Research Center in Madison, WI. F_1_ seeds were harvested from the B901 parent as the female and germinated in 2013. Four random F_1_ plants were selected and designated as numbers BS1, BS3, BS4, and BS7. The following four pseudo-F_2_ crosses were made in 2013: BS1 × BS7 (318 genotypes), BS7 × BS1 (98 genotypes), BS3 × BS4 (114 genotypes), and BS4 × BS3 (58 genotypes). This created a total of 588 tetraploid genotypes. The initial cross was made between upland and lowland ecotypes to generate as much allelic variation as possible in flowering time, based on phenotypic differentiation between the two ecotypes. The second set of crosses was made to generate segregation at all relevant loci that might have been homozygous in the two original parents, which would then have been non-segregating heterozygotes in the F1 individuals.

Newly germinated seedlings were transplanted to containers (2.5 cm diameter) and grown under natural and supplemental light in a greenhouse. The tillers were split multiple times so that each F_2_ genotype contained four tillers. Parents and grandparents were similarly propagated to serve as controls. After individual tillers began producing new tillers, they were separated into two groups: one or two clones of each F_2_ genotype assigned to each of two locations, DeKalb, IL (41.77 N) and Lafayette, IN (40.43 N). Seedlings were transplanted in July 2014 and arranged in an augmented experimental design with 10 blocks at each location, similar to the design that was previously described (Casler et al., 2015). A total of 588 F_2_ genotypes with one or two clonal replicates each were randomly assigned across the 10 blocks with all parents and grandparents included in each block as controls. The spacing between adjacent plants was 0.9 m. Plants were fertilized with 100 kg N ha^-1^ in early spring for 2015 and 2016. Weed control was maintained by applying a pre-emergent herbicide. Spot treatments and weeding were employed throughout the growing season to ensure weed pressure was minimal.

### Phenotyping

Heading and anthesis dates were recorded for 588 switchgrass genotypes for both locations in 2015 and 2016. Heading date was determined when 50% of the tillers had emerged panicles, while anthesis was defined by the presence of one floret flowering for 50% of the tillers. Analysis of variance was completed in PROC MIXED with blocks as the only random effect (SAS Institute, Version 9.1, Cary, NC, United States). The augmented design was implemented by using the control genotypes within each block as adjustment factors for block-to-block variation, treating them as random covariates within the mixed model analysis. A completely random effects model was used for estimating broad-sense heritability. Heritability (H^2^) was estimated as follows: H^2^ = σ^2^_g_/(σ^2^_g_ + σ^2^_ge_/l + σ^2^_e_/rl), where σ^2^_g_ is the variance component for genotype, σ^2^_ge_ for genotype-by-environment, σ^2^_e_ for error, r number of replications, and l is the number of environments (Sukumaran et al., 2016). Least squares means for both heading and anthesis dates were generated for each location separately as well as the combined location and year.

### Genotyping

The four pseudo-F_2_ populations were genotyped using exome capture sequencing as described previously in Evans et al. (2015) and adapted in Tornqvist et al. (2018) to produce 101-nucleotide paired-end reads, with an average of 12 M reads per sample (**Supplementary Table S1**). Reads were initially examined for quality using FastQC 0.11.5^1^ and trimmed using CutAdapt v1.9.1 (Martin, 2011). Reads were aligned to the *Panicum virgatum* genome (v.1.1 hardmasked)^2^ using Bowtie v0.12.7 (Langmead et al., 2009) allowing only uniquely mapping reads with a single mismatch in the seed region, and a minimum read length of 35 nucleotides. Reads were sorted and indexed with Samtools v0.1.19 (Li et al., 2009). Pileup files were generated using Samtools (v0.1.19) mpileup with BAQ disabled and map quality adjustment disabled. An initial set of single nucleotide polymorphisms (SNPs) were called using read data only at SNP loci identified previously in a northern switchgrass diversity panel of 537 individuals (Evans et al., 2015) and filtered to remove any alleles not present in the original dataset.

All raw SNPs were filtered using a custom script (**Supplementary R scripts Data.processing**) in the R programming language (R Core Team, 2014), with a call rate > 0.8 and sequencing depth > 1.6 (set from the expected depth for call rate = 0.8, according to a Poisson distribution). SNP markers were filtered for MAF > 0.05, based on simple genotype calls (directly based on co-occurrence of polymorphic reads), and genotype probabilities (as estimated from the expectation-maximization algorithm of Martin et al., 2010). Markers were retained if they satisfied the aforementioned filtering criteria in all four segregating families. After marker filtering, markers were imputed with the expectation-maximization algorithm of Poland et al. (2012) setting imputed values < 0 and > 2 to 0 and 2, respectively (**Supplementary R scripts Imputation**). After imputation, we performed a chi-squared test to test for Hardy-Weinberg equilibrium (HWE) and discarded markers for which the p-value was <10^-5^ in any of the four populations.

### Genome-Wide Association Analysis

Principal component analysis (PCA) and genomic kinship (K) were calculated using TASSEL 5.0 software, with centered IBS method for K (Bradbury et al., 2007). Quantile–quantile (Q-Q) plots for model comparisons of simple linear (S), PCA, genomic kinship (K), and PCA + K across traits were generated using ‘qqman’ package in R (R Core Team, 2014), and the best fit model was selected for association analysis of each trait. Associations between SNPs and heading or anthesis dates were analyzed using the mixed linear model (MLM) in TASSEL 5.0 software (Bradbury et al., 2007) with the following data set: (1) across 2 years in each location; (2) across two locations and 2 years; and (3) across two locations in each year. Associations were considered to be significant only at a *P*-value lower than 0.05/N, where N was 37,901 SNPs.

### Candidate Gene Identification

Using the *P. virgatum* genome assembly v.1.1 (DOE-JGI)^3^, candidate genes containing SNPs or adjacent to SNPs extended to 10-, 20-, 30-, and 50-kb region were identified. For genes on unanchored contigs in the *P. virgatum* genome, we predicted the genomic location based on homology to the *Setaria italica* (Bennetzen et al., 2012) and *Sorghum bicolor* (Paterson et al., 2009) genomes using the PHYTOMINE tool in PHYTOZOME (Goodstein et al., 2012). Sequences were acquired from the National Center for Biotechnology Information online nucleotide database. The BLAST and Phytomine feature were also employed to gain further information regarding sequence similarity and putative function of genes identified. The protein sequences of the published *Arabidopsis thaliana* FT (Accession AB027505) was used to search for FT genes orthologs in switchgrass (v1.1) using BLASTP program with an *E*-value of 1E-5.

### Gene Expression by Quantitative Real-Time RT-PCR

Based on heading and anthesis dates, two genotypes of early flowering (3 and F_2_ individual 7071) and two genotypes of late flowering (1 and F_2_ individual 7055) were selected for examining gene expression profile using qRT-PCR. Phytomer tissues were collected on May 8th of 2017, representing V2-V3 stages of vegetative growth. The phytomer was defined as a node, the leaf at the node, a lateral bud, and an internode (Buck-Sorlin, 2013). Each sample consisted of three tillers per plant and had three replicates for each genotype. Briefly, total RNA was isolated using a Direct-zol^TM^ RNA MiniPrep Kit (Zymo Research Corp., Irvine, CA, United States) and then reverse transcription was performed with an iScript ^TM^ cDNA Synthesis Kit (Bio-Rad, Hercules, CA, United States). A volume of 10 μL mixture was used for all qPCRs reaction containing 1 μL of cDNA, the relevant primers, and iTaq Universal SYBR^®^ Green (Bio-Rad, Hercules, CA, United States) in Mx3000P qPCR system (Agilent Technologies, Santa Clara, CA, United States), with reaction for 10 min at 95°C followed by 40 amplification cycles of 10 s at 95°C, 30 s at 55°C, and 30 s at 72°C. Primer sequences for target genes and for switchgrass housekeeping gene of elongation factor 1-alpha (*eEF-1α*) (Gimeno et al., 2014) were listed in **Supplementary Table S2**. The method of 2^-ΔΔC_T_^ (Livak and Schmittgen, 2001) was used to calculate the relative expression level among early and late flowering genotypes. The analysis included three biological replicates and three technical replicates for each sampling time.

### Data Availability

Raw reads for pseudo-F_2_ populations have been deposited in the National Center for Biotechnology Information Sequence Read Archive under BioProject ID (PRJNA450338). The initial SNP calls based on the positions identified in Evans et al. (2015) are available on the Dryad Digital Repository under doi (to be released upon publication). The final filtered SNP matrices used in the analyses were shown in **Supplementary Table S3**.

## Results

### Phenotypic Variation

Heading and anthesis dates were recorded in 2015 and 2016 at Lafayette and DeKalb locations. Across 2 years, heading ranged from 177 to 211 days of the year and anthesis ranged from 193 to 245 days of the year at Lafayette, while heading ranged from 179 to 252 and anthesis varied from 186 to 261 days of the year at DeKalb (**Table 1**). Overall, there was a 34- and 73-day difference in heading and a 52- and 75-day difference in anthesis at the Lafayette and DeKalb locations, respectively. There were significant variation with respect to genotype, location, year, genotype × year, genotype × location, and genotype × location × year (**Table 2**). Broad-sense heritability was high enough for heading date (0.73) and anthesis date (0.74). Thus, GWAS analyses were conducted separately for each location and year and combined across locations and/or years only when the results were homogeneous. The trends in the relationship between heading or anthesis date and accumulated growing degree (GDD) were very similar in year 2015 and 2016 (**Supplementary Figure S1**). There was also a strong linear correlation (*r* > 0.99) between heading or anthesis date with GDD across years, genotypes, and locations (**Supplementary Figure S1**). Thus, day of year was chosen for calculating heading and anthesis dates and subsequently used for GWAS analysis.

**Table 1:** Range and mean values for heading and anthesis dates in Lafayette, IN and DeKalb, IL across 2015 and 2016 years.

Location	Trait	Range (day of the year)	Mean (day of the year)
Lafayette	Heading	177–211	187
	Anthesis	193–245	218
DeKalb	Heading	179–252	203
	Anthesis	186–261	233


**Table 2:** Mixed model analysis of variance for fixed effects for heading and anthesis dates in Lafayette, IN and DeKalb, IL across two years of 2015 and 2016.

		*df*	Type III SS	*F*-value	Significance
Heading	Year (Y)	1	31521	609.74	^∗∗∗^
	Location (L)	1	103081	1993.99	^∗∗∗^
	Y × L	1	38258	740.08	^∗∗∗^
	Genotype (G)	587	96817	3.19	^∗∗∗^
	G × Y	566	39223	1.34	^∗∗^
	G × L	352	24411	1.34	^∗∗∗^
	G × L × Y	318	26457	1.61	^∗∗∗^
Anthesis	Year (Y)	1	34633	797.53	^∗∗∗^
	Location (L)	1	115341	2656.02	^∗∗∗^
	Y × L	1	57146	1315.94	^∗∗∗^
	Genotype (G)	583	112532	4.44	^∗∗∗^
	G × Y	544	50519	2.14	^∗∗∗^
	G × L	346	31693	2.11	^∗∗∗^
	G × L × Y	257	21834	1.96	^∗∗∗^


*^∗^Significant at 0.05 significance level. ^∗∗^Significant at 0.01 significance level. ^∗∗∗^Significant at 0.001 significance level.*

### Genotyping and Principal Component Analysis

After filtering raw SNPs and fitting segregation patterns, a total of 37,901 SNPs was generated across all populations. PCA across 588 genotypes showed differentiation among the four sibling populations (**Supplementary Figure S2**). Two distinct groups were formed in the first principal component (PC1) separating sibling populations based on reciprocal crosses (BS1 × BS7 and BS7 × BS1; BS3 × BS4, and BS4 × BS3) (**Supplementary Figure S2**).

### GWAS for Heading and Anthesis

**FIGURE 1:**
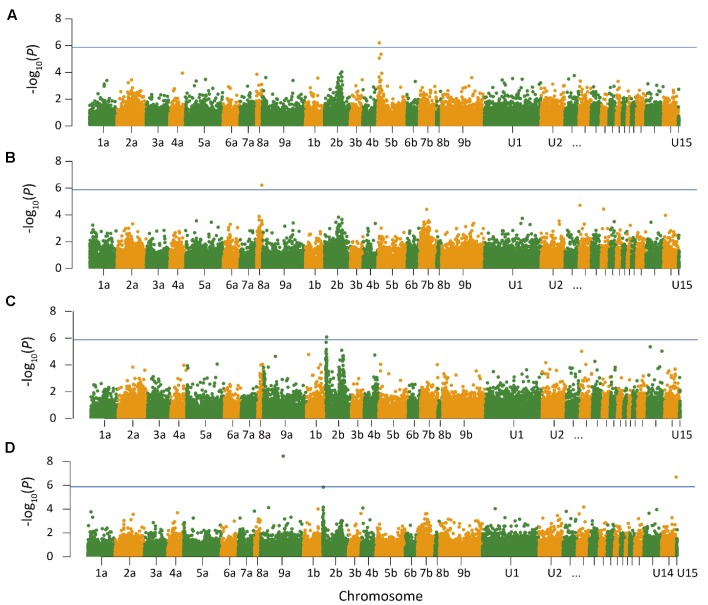
Manhattan plot of genome wide association analysis of switchgrass (*Panicum virgatum*) for Lafayette, IN across year 2015 and 2016 **(A)**, heading for Lafayette, IN and DeKalb, IL across year 2015 and 2016 **(B)**, heading for Lafayette, IN and DeKalb, IL in year 2016 **(C)**, and anthesis for Lafayette, IN and DeKalb, IL in year 2016 **(D)**. Lines indicate threshold for significant single nucleotide polymorphism. The *x*-axis indicates the physical position of the SNPs on the 18 switchgrass chromosomes; positional information in U1–U15 refer to unanchored scaffolds and contigs.

Quantile–quantile plots verified the adequate model for controlling false positives for GWAS of heading and anthesis dates (**Supplementary Figure S3**). Comparisons of observed and expected -log_10_ (*P*) showed that PCA plus K model was most suitable for analyzing SNP-trait associations. GWAS was performed in three ways to test for associations. First, GWAS was performed for heading and anthesis dates across 2 years for the Lafayette and DeKalb locations separately. The Lafayette location had a significant SNP identified on chromosome 5b for heading (**Figure 1**), but no significant SNPs were identified for DeKalb location for both traits. Across both locations and both years, one significant SNP for heading date was identified on chromosome 8a (**Figure 1**). GWAS was also completed for each year at both locations. Data from 2015 did not yield any significant SNPs across both locations, but year 2016 data contributed to three significant SNPs (**Figure 1**). The year 2016 combined for both locations had a significant SNP for heading on chromosome 2b, while one significant SNP for anthesis date on chromosome 9a and one SNP for anthesis date in the unanchored region 14 were also identified (**Figure 1**).

### GWAS and Candidate Genes for Heading and Anthesis

#### Heading in Lafayette Across Two Years

**FIGURE 2:**
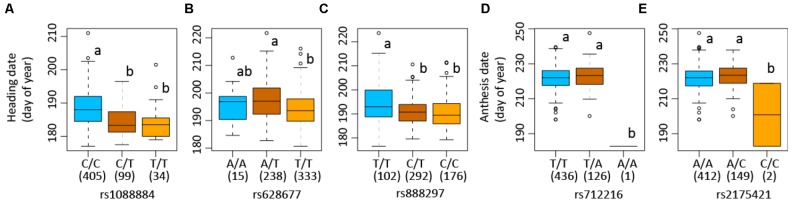
Allelic variations of single nucleotide polymorphisms (SNPs) associated with heading and anthesis dates in switchgrass (*Panicum virgatum*). Boxplots show heading or anthesis date in days of the year. Numbers of each genotype are indicated in parenthesis in box plots. Numbers following “rs” are SNP position. Plots show samples used in genome-wide association studies in which association is detected: Lafayette, IN across year 2015 and 2016 **(A)**, heading for Lafayette, IN and DeKalb, IL across year 2015 and 2016 **(B)**, heading for Lafayette, IN and DeKalb, IL in year 2016 **(C)**, and anthesis for Lafayette, IN and DeKalb, IL in year 2016 **(D)**. Columns with the same letter were not significantly different at *P* < 0.05.

The SNP on chromosome 5b for heading was located within the gene *Pavir.Eb00235*, which encodes a brassinosteroid signaling regulator that regulates transcription (**Table 3**). Genotypes with homozygous C:C alleles at SNP position rs1088884 had significantly later heading date than those carrying heterozygous T:C and homozygous T:T for early heading (**Figure 2**). Several genes of interest were located within 30 kb (**Table 4**), including a homolog of FLOWERING LOCUS T (FT), homologs of ARABIDOPSIS NOD26-LIKE INTRINSIC PROTEIN (AtNLM1;2), and a homolog of LIGHT REGULATED ZINC FINGER PROTEIN (LZF1).

**Table 3:** Significant SNPs for heading and anthesis dates identified by GWAS for Lafayette and Lafayette/DeKalb location.

SNP	Chr.	Position	Trait	Sample set	Year	No. of genotypes	*P-*values	Nearest gene ID
rs1088884	5b	3772986	Heading	Lafayette	2015/2016	538	6.15E-07	*Pavir.Eb00235*
rs628677	8a	51715776	Heading	Lafayette/DeKalb	2015/2016	586	6.03E-07	*Pavir. Ha01813*
rs888297	2b	1737687	Heading	Lafayette/DeKalb	2016	570	8.03E-07	*Pavir.* *Bb00124*
rs712216	9a	55104939	Anthesis	Lafayette/DeKalb	2016	563	3.50E-09	*Pavir.Ia02791*
rs2175421	Undefined 14	49102800	Anthesis	Lafayette/DeKalb	2016	563	6.33E-08	*Pavir.J40827*


**Table 4:** Candidate gene associations for heading and anthesis dates identified by GWAS for Lafayette and DeKalb for 2015 and 2016.

Distance (kb)	Switchgrass gene	Chr	*A. thaliana* homolog	*O. sativa* homolog	Function	Trait	Location	Year
Inside	*Pavir.Eb00235*	5B	AT1G78700.1	Os01g10610.1	BES1/BZR1 homolog 4	Heading	Lafayette	2015/2016
	*Pavir.Ha01813*	8A	AT5G52340.1	Os11g05880.1	Exocyst subunit exo70 family protein A2	Heading	Lafayette and DeKalb	2015/2016
10	*Pavir.Eb00236*	5B	AT4G18910.1	Os05g11560.1	NOD26-like intrinsic protein 1;2	Heading	Lafayette	2015/2016
	*Pavir.Eb00237*	5B	AT1G65480.1	Os01g10590.1	Flowering Locus T	Heading	Lafayette	2015/2016
	*Pavir.Ha01814*	8A	AT2G34320.1	Os03g30510.1	Polynucleotidyl transferase	Heading	Lafayette and DeKalb	2015/2016
	*Pavir.Ha01812*	8A	AT5G22870.1	Os11g05870.1	Late embryogenesis abundant	Heading	Lafayette and DeKalb	2015/2016
	*Pavir.Ia02791*	9A	AT1G50200.1	Os10g10244.1	Alanyl-tRNA synthetase	Anthesis	Lafayette and DeKalb	2016
	*Pavir.Ia02790*	9A	AT5G31412.1	Os01g52430.1	HAT transposon superfamily protein	Anthesis	Lafayette and DeKalb	2016
20	*Pavir.Bb00125*	2B	AT5G01220.1	Os07g01030.1	Sulfoquinovosyldiacylglycerol 2	Heading	Lafayette and DeKalb	2016
	*Pavir.Bb00123*	2B	AT2G39890.1	Os07g01090.1	Proline transporter 1	Heading	Lafayette and DeKalb	2016
	*Pavir.Ha01815*	8A	AT5G24470.1	Os11g05930.1	Pseudo-response regulator 5 (PRR5)	Heading	Lafayette and DeKalb	2015/2016
	*Pavir.Ha01810*	8A	AT3G11660.1	Os11g05860.1	NDR1/HIN1-like 1	Heading	Lafayette and DeKalb	2015/2016
	*Pavir.Ha01817*	8A	AT3G44190.1	Os11g05970.1	FAD/NAD(P)-binding oxidoreductase	Heading	Lafayette and DeKalb	2015/2016
30	*Pavir.Eb00232*	5B	AT1G53380.2	Os01g10680.2	Plant protein of unknown function	Heading	Lafayette	2015/2016
	*Pavir.Eb00238*	5B	AT1G78600.2	Os01g10580.1	Light-regulated zinc finger protein 1	Heading	Lafayette	2015/2016
	*Pavir.Ha01819*	8A	AT1G06140.1	Os11g05980.1	Pentatricopeptide repeat (PPR)	Heading	Lafayette and DeKalb	2015/2016
	*Pavir.Ha01820*	8A	AT3G11670.1	Os11g05990.1	UDP-Glycosyltransferase	Heading	Lafayette and DeKalb	2015/2016
	*Pavir.Ha01808*	8A	AT5G44720.2	Os09g38772.1	Molybdenum cofactor sulfurase	Heading	Lafayette and DeKalb	2015/2016
	*Pavir.Bb00120*	2B	AT3G09220.1	Os07g01110.1	Laccase 7	Heading	Lafayette and DeKalb	2016


#### Heading at Lafayette and DeKalb Across Two Years

The SNP on chromosome 8a for heading at Lafayette and DeKalb across both years was located within *Pavir.Ha01813*, an exo70 family protein subunit, which functions in the production of an octameric protein implicated in tethering secretory vesicles to the plasma membrane (**Table 3**). Genotypes with homozygous A:A at SNP position rs628677 had the same heading dates compared to homozygous T:T (**Figure 2**). Several genes of interest were identified within 20 kb (**Table 4**). Notable homologs included ARABIDOPSIS PSEUDO-RESPONSE REGULATOR 5 (APPR5/PRR5) and UDP-Glycosyltransferase.

#### Heading at Lafayette and DeKalb in 2016

Identification of candidate genes for heading at both locations for 2016 was expanded to 50 kb due to the number of uncharacterized genes surrounding significant SNPs. The significant SNP on chromosome 2b for heading was located within *Pavir.Bb00124*, a gene with unknown function. Genotypes with homozygous alleles T:T at SNP position rs888297 had significantly later heading than those carrying heterozygous T:C and homozygous C:C for early heading (**Figure 2**). There were four genes of interest found within the 50 kb region identified by GWAS, including a homolog of PROLINE TRANSPORTER 1 (PRO1), a homolog of serine/threonine-protein kinase WNK, and a homolog of SULFOQUINOVOSYLDIACYL GLYCEROL 2 (SQDG2) (**Supplementary Table S4**).

#### Anthesis at Lafayette and DeKalb in 2016

The SNP on chromosome 9a associated with anthesis at both locations was located within the gene *Pavir.Ia02791* that encodes ALANYL-tRNA SYNTHETASE (ALATS) (**Table 3**). At SNP position rs712216, genotypes with homozygous alleles T:T and heterozygous T:A had substantially later anthesis dates than the one genotype carrying homozygous alleles A:A (**Figure 2**). A gene encoding a homolog of a hAT transposon superfamily protein was identified within 10 kb, and a homolog of ribonuclease H-like superfamily protein and a homolog of a RNA binding family protein were identified within the 50 kb region (**Table 4** and **Supplementary Table S4**). These genes primarily were related to nucleotide binding or protein dimerization, which could interact with developmental processes that control flowering, but currently, the function of these proteins in relation to floral development is not well understood. The SNP position rs2175421 in an undefined region was also associated with anthesis at both locations. *Pavir.J40827* encoding ADP-ribosylation factor-like factor was identified in this region (**Supplementary Table S4**). Genotypes with homozygous alleles A:A and heterozygous A:C had later anthesis dates than those carrying homozygous alleles C:C (**Figure 2**).

#### Gene Expression in Genotypes Contrasting With Flowering Time

**FIGURE 3:**
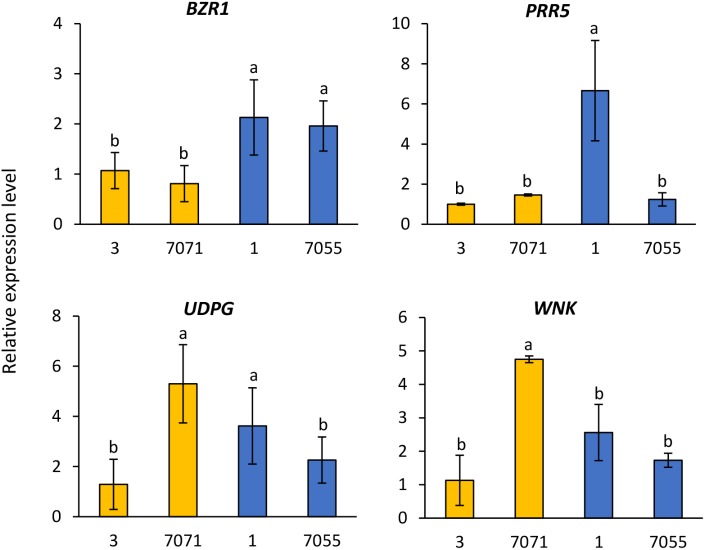
Relative gene expression level in the early flowering genotypes (3 and 7071) and late flowering genotypes (1 and 7055). The expression data were normalized relative to early genotype (3). Columns with the same letter were not significantly different at *P* < 0.05. BES1/BZR1, Brassinosteroid signaling positive regulator; PRR5, PSEUDO-RESPONSE REGULATOR 5; UDPG, UDP-Glycosyltransferase; WNK, Serine/threonine-protein kinase WNK.

The expression of four candidate genes *BZR1, PRR5, UDPG*, and *WNK* was analyzed in two early flowering genotypes (3 and 7071) and two late flowering genotypes (1 and 7075) (**Figure 3**). Relative to the early flowering genotype 3 and 7071, expression levels of *BZR1* were significantly higher in the two flowering genotypes (**Figure 3**). The higher expression of *PRR5* was also observed in the late flowering genotype 1, compared to other three genotypes (**Figure 3**). The early flowering genotype 7071 and late genotype 1 had higher expression of *UDPG*, while 7071 showed higher expression of *WNK* (**Figure 3**).

## Discussion

Significant interactions for heading and anthesis dates involving genotypes, locations, and years may have contributed to variability in GWAS results. Five significant SNPs at multiple loci on chromosome 5b, 8a, 2b, 9a, and undefined region of 14 were associated with either heading or anthesis date. These signals were detected in Lafayette location across 2 years, Lafayette and DeKalb locations across 2 years, and two locations in year 2016, but no signals were detected in year 2015. The higher average temperatures, GDD and precipitation from April to September across two locations observed in 2016 than in 2015 may have contributed to variations of growth and flowering time between the 2 years. Moreover, 2016 was the second year after planting and the grass plants were more established than year 2015, which may play a role in phenotypic variation. Comparing two locations, one signal was found in Lafayette across 2 years, but not in DeKalb. Similarly, the higher average temperatures and GDD from April to September in Lafayette than DeKalb may cause variation of growth and possibly lead to delayed flowering time at DeKalb. There were fewer signals detected for anthesis than heading in this study. Some genotypes with heading emergence but did not flower at the end of growing season. This may have influenced signal identifications related to anthesis.

It is worth mentioning that QTL mapping for flowering time has been performed using one of the four pseudo-F_2_ populations (BS1 × BS7) and same phenotypic data for this particular population in this study. Interestingly, 9 QTLs have been detected including one for heading and one for anthesis on chromosome 2b in Dekalb, 2016 and one for heading on chromosome 8a across Lafayette and DeKalb locations and 2 years of 2015 and 2016 (Tornqvist et al., 2018). Although these QTLs on chromosome 2b and 8a were not the same one identified in this study, all signals detected through GWAS and QTL mapping could be important targets for elucidating genetic control of flowering time in switchgrass. In addition, QTL mapping identified one signal on chromosome 2a separately for each location and year and combined across locations and/or years, but was not found in this study. Within this QTL region on chromosome 2a, homologs of flowering time genes were identified such as PSEUDO RESPONSE REGULATOR 5 (PRR5), SUPPRESSOR OF FRIGIDA 4, and APETALA 1, which are involved in the circadian clock, vernalization, and floral meristem identity, respectively (Tornqvist et al., 2018).

Genetic control of flowering has also been reported in Setaria, a panicoid grass closely related to switchgrass (Mauro-Herrera et al., 2013). Through analysis of flowering time of 182 F7 recombinant inbred lines developed from a cross between foxtail millet (*Setaria italica*) and its wild relative green foxtail (*Setaria viridis*) (Wang et al., 1998; Bennetzen et al., 2012), a total of 16 QTLs were deteced in eight trials conducted in greenhouses, field and growth chambers at different locations (Mauro-Herrera et al., 2013). Underlying QTL regions, flowering pathway genes were identified from rice (*Oryza sativa*), maize (*Zea mays*), sorghum, and *Arabidopsis* including PRR95, PRR59, GI involved in circadian clock and CONSTANS involved in the photoperiod pathway. Compared to the previous and current studies in switchgrass, the results in Setaria supported that some flowering genes such as PRR5 could play an important role in regulating flowering time across a range of grass species and other environmental factors.

Candidate genes related to plant growth and flowering were identified within 50 kb of significant SNPs. Such genes included BES1/BZR1 homolog 4, FLOWERING LOCUS T (FT), pseudo-response regulator 5 (PRR5), light-regulated zinc finger protein 1, UDP-Glycosyltransferase, hAT transposon superfamily protein, helix-loop-helix DNA-binding protein, and serine/threonine-protein kinase WNK (**Table 4** and **Supplementary Table S4**). The significant SNP related to heading on chromosome 5b was inside gene *Pavir.Eb00235*, which was a homolog of *BES1/BZR1* encoding a brassinosteroid signaling regulator. This SNP was deemed significant at Lafayette location across 2015 and 2016 (**Table 4**). Brassinosteroids (BRs) are a class of steroidal hormones essential for plant growth and development, including regulating flowering time (Li et al., 2010). BZR1/BES1 can bind directly to the promoter regions of the BR biosynthetic genes, *CPD* and *DWF4*, and inhibit their expression (Tanaka et al., 2005). BR biosynthetic mutants of *CPD* and *DWF4* had delayed flowering time (Li et al., 2010). In this study, higher expression levels of *BZR1* shown in the two late flowering genotypes of switchgrass compared to the early types supported that increased expression of this gene could inhibit BR biosynthetic genes, thus delaying flowering. These results support previous findings from expression analysis of flowering time genes in switchgrass (Tornqvist et al., 2017).

Candidate gene *Pavir.Ha01815* that encodes PRR5 was identified on chromosome 8a within 20 kb of SNP related to heading across Lafayette and DeKalb locations and years (**Table 4**). Through linkage mapping analysis in one of the populations used in this study, one PRR5 on chromosome 2a related to heading and anthesis date was detected in Lafayette or DeKalb location (Tornqvist et al., 2018). Although the two *PRR5* were located on different chromosomes, the results from GWAS and QTL mapping suggest that *PRR5* plays a role in regulating flower time. PRR5 has been shown to modulate light input into the circadian clock (Nakamichi et al., 2005). In *A. thaliana*, PRR5 regulates the period of free-running rhythms of certain clock-controlled genes including CIRCADIAN CLOCK ASSOCIATE 1 (CCA1) and ARABIDOPSIS PSEUDO-RESPONSE REGULATOR 1 (APRR1) (Kamioka et al., 2016). The PRR5 mutant of *A. thaliana* showed late flowering under continuous light, late flowering under long days, and early flowering under short days (Yamamoto et al., 2003; Nakamichi et al., 2007; Niinuma et al., 2008). However, *PRR5*-overexpressing transgenic lines of *Arabidopsi*s flowered earlier than the wild-type plants under both long and short day conditions (Sato et al., 2002). In barley (*Hordeum vulgare*), *PRR59* and *PRR95*, homologs of *At PRR5* or *AtPRR*9, respectively, exhibited higher expression abundances in late flowering genotypes compared to the early flowering genotypes under long day conditions (Campoli et al., 2012). The transcriptomic analysis showed that expression of *PRR5* was either up- or down-regulated or remained unchanged in early or late flowering genotypes at different growth stages of switchgrass (Tornqvist et al., 2017). In this study, one late flowering genotype had a much higher expression of *PRR5* relative to other early and late flowering genotypes. Collectively, it appears that expression of *PRR5* genes varies across plant species, genotypes and environmental conditions.

The candidate gene *Pavir.Ha01820*, a homolog of UDP-Glycosyltransferase was identified on chromosome 8a within 30 kb of a SNP associated with heading date across the Lafayette and DeKalb locations and years (**Table 4**). Glycosyltransferases (GTs) are the enzymes for the glycosylation of plant compounds (Bowles et al., 2005). GTs might play an important role in the maintenance of cell homeostasis and regulation of plant growth and defense responses (Jones and Vogt, 2001; Lim and Bowles, 2004). An *Arabidopsis* mutant of *UGT87A2*, encoding a putative family 1 GT, had delayed flowering time, while overexpression of *UGT87A2* caused much earlier flowering than the mutant (Wang et al., 2012). They further verified that *UGT87A2* regulated flowering time via the flowering repressor FLC (Wang et al., 2012). The transcriptomic analysis indicated up, down, or unchanged expression level of *UGPD* in early or late flowering switchgrass genotypes in response to different growth stages (Tornqvist et al., 2017). Our results supported that expressions of *UGPD* were not consistent across genotypes with higher level found in one early flowering (7071) and one late flowering genotype (1) of switchgrass.

Within 50 kb of a SNP on chromosome 2b related to heading date, *Pavir.Bb00118*, a homolog of serine/threonine-protein kinase WNK, was identified across Lafayette and DeKalb locations in 2016 (**Supplementary Table S4**). Protein kinases play important roles in controlling diverse cellular processes (Manning et al., 2002). In *Arabidopsis, AtWNK1, AtWNK2, AtWNK4*, and *AtWNK6* seem to be under the control of the circadian clock (Nakamichi et al., 2002). For instance, *AtWNK1* interacts with APRR3 and phosphorylates the APRR3 component of APRR1 /TOC1 in plants (Nakamichi et al., 2002). Further studies showed that *AtWNK2, AtWNK5*, and *AtWNK8* mutants caused early flowering, while in contrast, *AtWNK1* mutant delayed flowering time (Wang et al., 2008). In this study, gene *Pavir.Bb00118* is a homolog of *AT1G64625*, also named FEHLSTART (FST) and WNK10 in *Arabidopsis* (Li et al., 2015). The *fst-1* mutant had normal vegetative growth and floral organ development, but showed low fertility and shorter siliques with fewer seeds (Li et al., 2015). Higher expression of *WNK* in one early flowering genotype demonstrated that WNK could be involved in the early flowering response. The transcriptomic analysis indicated up- or down-regulated expression level of *WNK* in early or late flowering switchgrass genotypes (Tornqvist et al., 2017). These results suggest that expression of *WNK* varies with growth stage and genotype. In addition, the transcript levels of ELF4, TOC1, CO, and FT were altered in *AtWNK* mutants, indicating that *WNK* genes regulate flowering time by modulating the photoperiod pathway (Wang et al., 2008).

Within 10 kb of an identified SNP on chromosome 5b, *Pavir.Eb00237*, a homolog of FLOWERING LOCUS T (FT) related to heading date was identified at Lafayette location across 2015 and 2016 (**Table 4**). FT is a key component of the photoperiodic pathway. In *A. thaliana*, the photoperiodic pathway acts through FT to promote floral induction in response to day length (Andrés et al., 2015). Allelic variation in the FT gene was associated with flowering time in natural or seminatural populations of perennial ryegrass (*Lolium perenne*) (Skøt et al., 2011). Gene *Pavir.J05082*, a homolog of the major flowering time regulator FT, was associated with early flowering in switchgrass natural populations (Grabowski et al., 2017). However, the transcriptomic analysis indicated very low expression levels of FT genes in early and low flowering switchgrass genotypes (Tornqvist et al., 2017). Similarly, we were not able to detect expression of FT through qRT-PCR in early and late flowering samples. By blasting against *Arabidopsis* FT family, we found a total of 47 FT genes in switchgrass genome (**Supplementary Table S5**). Further studies could verify the role of these genes in regulating flowering time.

## Conclusion

Plant flowering is regulated by a complex network of genetic and environmental signals. The work presented here elucidates the genetic mechanisms controlling heading and anthesis dates in four pseudo-F_2_ populations (two pairs of reciprocal crosses) of switchgrass through GWAS. Five significant SNPs were detected and associated with heading or anthesis dates, and candidate genes for light signaling, reproductive structures, circadian clock rhythm, and flowering time were identified. The results indicated genetic complexities (i.e., multiple regions/components) related to floral development. Future research could verify gene function associated with heading and anthesis development, which has great potential to enhance breeding programs aimed at germplasm improvement of switchgrass.

## Author Contributions

MT and C-ET collected the phenotypic data. MT and XZ performed the statistical and GWAS analysis. JE and CB performed the genotyping using exome-capture sequencing technique and raw SNP calling. MT, GR, and MS conducted further SNP filtering. C-ET, XZ, PG, GR, MS, CB, RD, JM, JV, and MC participated in interpreting the results and writing the manuscript. MC and YJ designed the experiments. MT and YJ led writing of the manuscript.

## Conflict of Interest Statement

The authors declare that the research was conducted in the absence of any commercial or financial relationships that could be construed as a potential conflict of interest.

## Footnotes

1 www.bioinformatics.babraham.ac.uk

2 phytozome.jgi.doe.gov

3 http://phytozome.jgi.doe.gov/
