# Supplementary material for: Genome-Wide Association Study in Pseudo-F2 Populations of Switchgrass Identifies Genetic Loci Affecting Heading and Anthesis Dates
Source: Front Plant Sci. 2018 Sep 13;9:1250. doi: 10.3389/fpls.2018.01250 (PMC6146286; doi:10.3389/fpls.2018.01250)
Supplement: Supplementary file 4 [file Presentation_1.pdf]

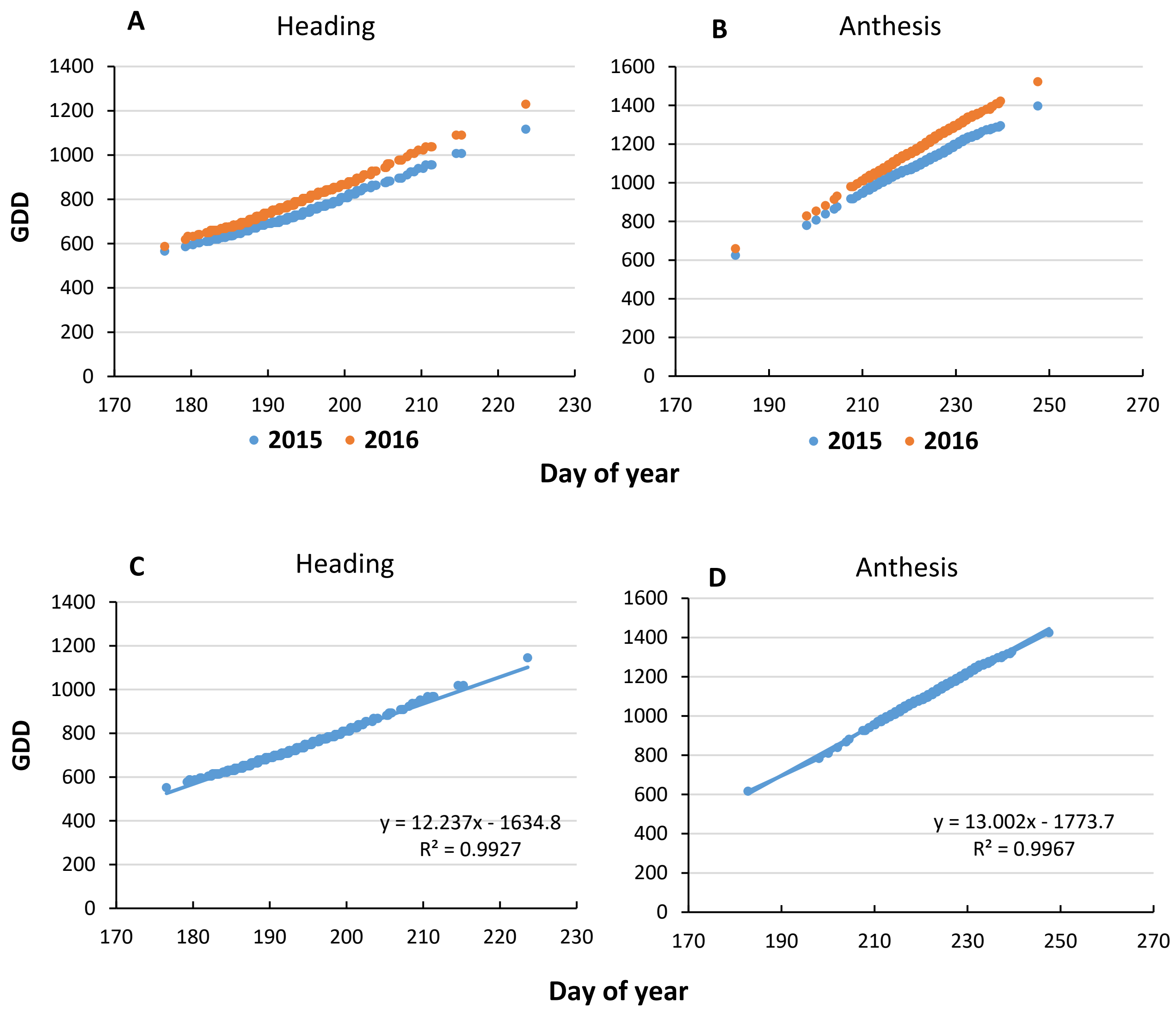

Supplemental Figure S1. Year comparisons between heading date (A) and anthesis date (B) and accumulated growing degree days (GDD) across locations of DeKalb, IL and Lafayette, IN and genotypes in 2015 and 2016. Relationships between heading date (C) and anthesis date (D) with accumulated growing degree days (GDD) across locations, genotypes and two years. Daily GDD was calculated as: [(maximum temperature + minimum temperature/2)-10°C]. GDD starts accumulating after five consecutive days with average temperature >10°C (Grabowski et al., 2017).

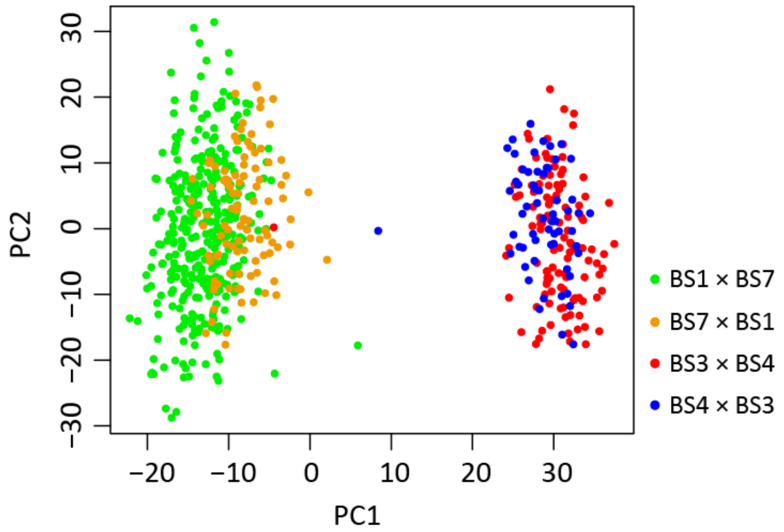

Supplemental Figure S2. Principal component analysis (PCA) of 588 switchgrass (*Panicum virgatum*) genotypes used for genome-wide association analyses at Lafayette, IN and DeKalb, IL. The individuals among the two crosses with reciprocals were separated into two distinct groups. BS1 × BS7 and BS7 × BS1S are reciprocal; BS3 × BS4, and BS4 × BS3 are reciprocal. Principal component 1 (PC1) is the x-axis and Principal component 2 (PC2) is the y-axis. PC1 is the separation of sibling populations.

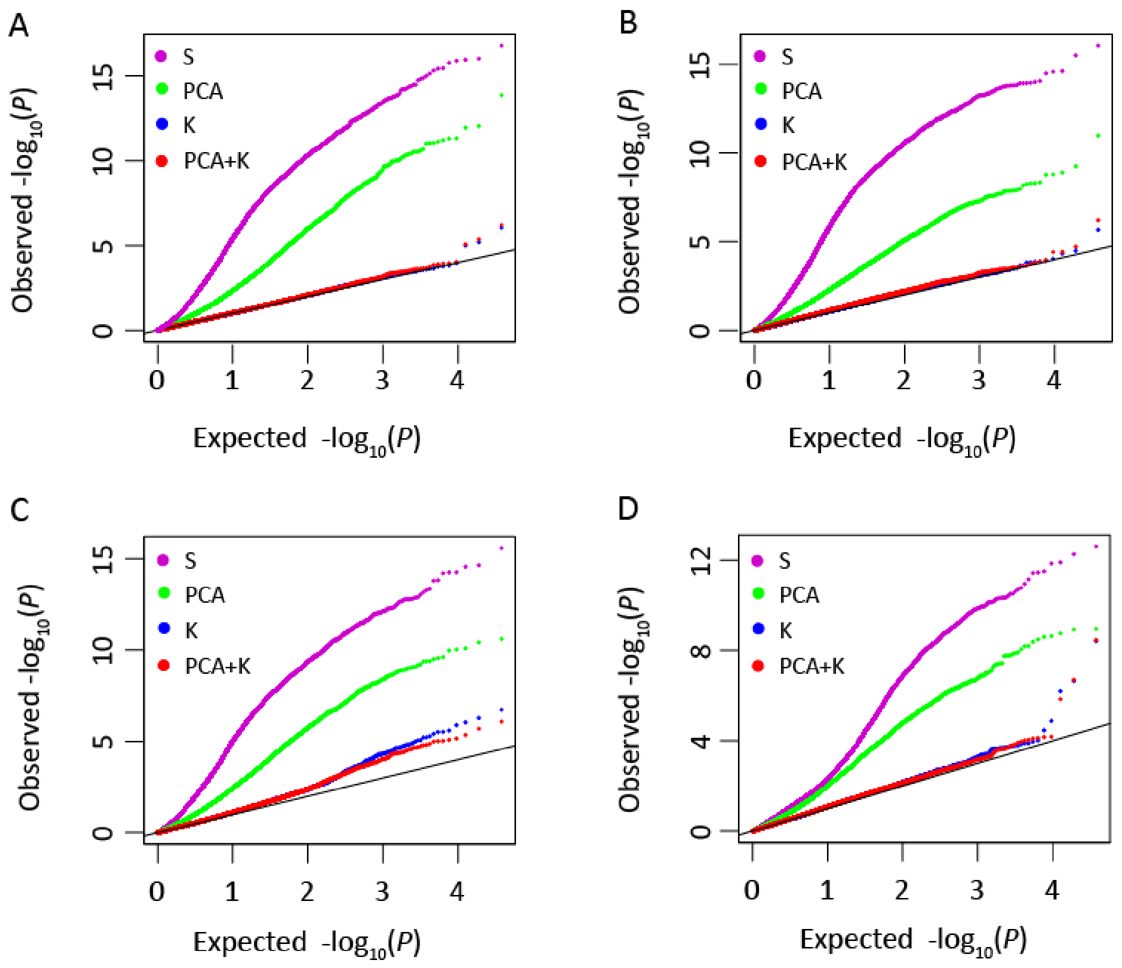

Supplemental Figure S3. Quantile-quantile (QQ) plots of switchgrass (*Panicum virgatum*) for model comparisons with heading for Lafayette, IN across year 2015 and 2016 (A), heading for Lafayette, IN and DeKalb, IL across year 2015 and 2016 (B), heading for Lafayette, IN and DeKalb, IL in year 2016 (C), and anthesis for Lafayette, IN and DeKalb, IL in year 2016 (D). The solid diagonal lines represent agreement between the observed and expected -log 10 (P). Color lines represent agreement between the observed and expected -log 10 (P) value for marker and trait associations analyzed with simple liner (S), principal component analysis (PCA), relative kinship (K), and PCA+K implemented model, respectively.
